# Concurrent functional-structural reorganization in brain networks of AVM patients: a functional and structural study

**DOI:** 10.3389/fneur.2025.1619226

**Published:** 2025-10-28

**Authors:** Qinghui Zhu, Heze Han, Li Ma, Ruinan Li, Zhipeng Li, Anqi Li, Haibin Zhang, Kexin Yuan, Chengzhuo Wang, Yukun Zhang, Hongwei Zhang, Yu Chen, XiaoLin Chen

**Affiliations:** ^1^Department of Neurosurgery, Beijing Tiantan Hospital, Capital Medical University, Beijing, China; ^2^Department of Neurosurgery, International Hospital, Peking University, Beijing, China

**Keywords:** cerebral arteriovenous malformations, brain network, resting-state functional MRI, diffusion tensor imaging, tract-weighted functional connectivity

## Abstract

**Background:**

Unruptured cerebral arteriovenous malformations (AVMs) generally do not cause focal neurological deficits, prompting limited investigation into potential neurological changes associated with them.

**Purpose:**

To determine whether AVMs exhibit combined functional and structural reorganization using resting-state functional MRI (rs-fMRI) and diffusion tensor imaging (DTI).

**Study type:**

Retrospective study.

**Population:**

44 AVM patients who underwent both rs-fMRI and DTI examinations as well as an equal number of age- and sex-matched healthy controls.

**Sequence:**

Functional alterations were assessed using amplitude of low-frequency fluctuation (ALFF) analysis and functional connectivity networks, while fiber alterations were examined through fractional anisotropy (FA) analysis and tract-weighted functional connectivity (TW-FC) analysis.

**Assessment:**

Functional alterations were evaluated by ALFF and functional connectivity networks, analyzed by neuroimaging specialists. Structural alterations were assessed through FA and TW-FC analysis, performed by experienced radiologists.

**Statistical tests:**

Independent two-sample *t*-test and the Mann– Whitney *U* test were used to analyze the continuous variables. Chi-squared test was used to test the categorical variables. We used permutation test with family-wise error correction while setting the statistical threshold of *p* < 0.05 at the cluster level. Two-tailed statistical significance was set at *p* < 0.05.

**Results:**

AVMs showed significant ALFF differences in 12 brain regions and altered functional connectivity networks compared to healthy controls (*p* < 0.05). Fiber connectivity and density were significantly reduced in AVM patients (*p* < 0.05). TW-FC analysis indicated significant differences across regions of interest (ROIs) between AVMs and healthy controls, suggesting integrated functional and structural reconfigurations (*p* < 0.05).

**Data conclusion:**

The study reveals significant functional and structural changes in AVM patients, particularly in the visual network (VN) and sensorimotor network (SMN). These alterations suggest compensatory mechanisms that may offset functional deficits, providing insights into AVM pathophysiology and potential strategies for optimizing treatment to mitigate functional impairments and promote recovery.

## Highlights

- There is a profound connection between arteriovenous malformations and a series of global brain changes in brain function and fiber reorganization.
- The concurrent functional and fiber connectivity changes between the Visual Network and the Sensorimotor Network in AVMs suggest the innate ability of the brain to organize compensatory mechanisms.
- The differences in Tract-Weighted Functional Connectivity can serve as non-invasive parameters for longitudinal monitoring of disease progression and systematic assessment of intervention efficacy.

## Introduction

Cerebral arteriovenous malformations (AVMs) are characterized by a network of abnormal blood vessels where the feeding arteries are directly connected to the draining veins, exhibiting high-flow, low-resistance hemodynamic features (1, 2). AVMs may rupture and cause bleeding, leading to symptoms such as seizures and loss of consciousness (3, 4). However, for unruptured AVMs, due to the rarity of symptoms related to focal neurological deficits (4, 5), there has been limited attention on whether AVMs cause neurological functional changes and fiber alterations.

Unruptured AVMs usually do not exhibit these symptoms, even when the lesion is located in eloquent areas (6, 7), which is different from brain tumors, ischemic stroke, and intracranial hemorrhage (8). One possible explanation is that AVM lesions are congenital, and potential functional reorganization in these regions (9, 10) distinguishes them from acquired conditions like brain tumors. There is ongoing discussion about whether the reorganization associated with AVMs occurs at a functional level or is based on structural changes. Some reports suggest that preoperative fiber changes in AVM patients may be related to their functional prognosis (11), indicating unique alterations in fiber function and supporting the hypothesis of brain reorganization in AVM patients. Given the findings from unimodal studies of structural and functional imaging may involve structure–function relationships, and magnetic resonance diffusion tensor imaging (DTI) is currently the only method capable of tracing white matter fiber tracts *in vivo* (12). Researchers have utilized DTI to observe the impact of AVM on fiber tracts. However, previous studies have primarily focused on the relationship between AVM and the surrounding fibers (13, 14), and there is insufficient reporting on the concurrent changes in function within the fiber connectivity regions.

Resting-state functional MRI (rs-fMRI) is a commonly used non-invasive imaging technique that has been widely applied in the study of various diseases, such as Alzheimer’s disease, stroke, mild cognitive impairment, and gliomas (15–18). Amplitude of low-frequency fluctuation (ALFF) analysis is a data-driven method in rs-fMRI that investigates regional spontaneous activity and measures the intensity of blood oxygen-level dependent (BOLD) signals in low-frequency oscillations within local brain regions (19). Changes in ALFF may represent underlying abnormalities in brain activity (20, 21). Previous studies have used rs-fMRI to explore the relationship between AVM and changes/reorganization in language function (5, 22). However, these studies have only observed the functional changes without delving into the causes and processes underlying these changes.

To delve deeper into the intricacies of brain reorganization in relation to AVMs, it is imperative to examine the potential associations between AVM and alterations in both neural function and fiber connectivity. We conducted a retrospective study of AVM patients who visited our hospital between 2020 and 2023, all of whom underwent both fMRI and DTI examinations. This study also included demographic and imaging data from healthy controls available in open databases for comparison. To our knowledge, this study is the first to simultaneously demonstrate differences in brain function and fiber tract alternations between AVM patients and normal brains.

## Methods

### Participants and study design

We recruited 44 patients who were diagnosed with brain AVM at the department of neurosurgery of our hospital between May 2020 and June 2023. The inclusion criteria were as follows: 1. Diagnosis of AVMs confirmed by radiological findings, 2. Patients who underwent presurgical fMRI and DTI. The exclusion criteria were as follows: 1. Prior rupture or intervention for AVM before admitting to our institution, 2. Diffused or trans-hemispheric lesions, 3. Patients with other neurological or psychiatric disorders, 4. Poor quality MRI detected after preprocessing. This study was approved by the institutional review board of Capital Medical University, according to the principles of the Declaration of Helsinki. All enrolled patients have provided informed consent. To ensure age/sex matching while addressing practical limitations in recruiting matched controls for specialized MRI protocols, age- and sex-matched healthy controls (HC) were obtained from The National Institute of Mental Health (NIMH) Intramural Healthy Volunteer Dataset (23) and The Single Individual volunteer for Multiple Observations across Networks (SIMON) MRI dataset (24). Due to the limited availability of clinical data on healthy individuals, this approach mitigates selection bias in our cohorts.

### MRI data acquisition

All MRI scans were obtained on the same Siemens 3 T MRI scanner with a 64-channel phased-array head coil. Structural brain images were obtained using three-dimensional magnetization-prepared acquisition with the following parameters: repetition time (TR) = 2,530 ms, echo time (TE) = 2.02 s, flip angle = 7°, matrix size = 256 × 256, slice thickness = 1.0 mm, 192 slices. Resing-state fMRI was obtained using an Echo Planar Imaging sequence with parameters: TR = 2020 ms, TE = 30.0 ms, flip angle = 90°, matrix size = 64 × 64, slice thickness = 3.5 mm, 33 slices. During the scan acquisition, the participants were asked to keep their eyes closed, not to think of anything and not to fall asleep. Diffusion MRI parameters: TE/TR = 91/ 9,800 ms, 2 mm isotropic resolution, 60 diffusion weighted encoding directions, b value = 3,000 s/mm^2^. All MRI were verified by two credentialed senior neuroradiologists (YKZ and YZ).

### Surface-based analysis

The surface-based analysis was performed using the toolbox for Data Processing & Analysis for Brain Imaging on Surface (DPABISurf) (25), which is based on fMRIPrep (26), FreeSurfer (27), ANTs (28), FSL (29), AFNI (30), SPM (31), dcm2niix (32), PALM (33), GNU Parallel (34), MATLAB (The MathWorks Inc., Natick, MA, US), Docker (https://docker.com) and DPABI (35). Use fMRIPrep for preprocessing and correcting head movements. Before group-level analysis, we applied harmonization to the DTI-derived metrics (FA, MD) and fMRI time series to account for scanner/site effects arising from variations in acquisition parameters (e.g., TR/TE) and site-specific noise characteristics. Afterwards, calculate the amplitude of low-frequency fluctuations and analyze the differences in brain regions between the two groups. Using brain regions with significant differential ALFF values as regions of interest (ROIs), the ROI time series and functional connectivity matrices were extracted.

### Diffusion-weighted imaging analysis

Data Processing & Analysis for Brain Imaging on Network on fiber (DPABIFiber) is a fiber tractography analysis toolbox based on DTI. The raw T1 and DTI data were preprocessed with QSIPrep (36), which integrates FSL and ANTs. Then, the tensors and the DTI metrics including apparent diffusion coefficient (ADC), fractional anisotropy (FA), axial diffusivity (AD), and radial diffusivity (RD) are derived from preprocessed DTI data using MRtrix3 and subsequently generate tract-based spatial statistics (TBSS) (37) results using FSL. After that, the QSIPrep is called again to reconstruct fiber tracts. Based on the constructed fiber tract, advanced analysis such as structural connectome matrix and seed-based structural connectivity with ROI could be further conducted. For seed-based structural connectivity analysis, we generated tracks for each ROI. Tract-weighted functional connectivity (TW-FC) (38) was constructed by performing element-wise multiplication between structural connectivity weights derived from probabilistic tractography of DTI data across ROIs, and functional connectivity matrices generated via Pearson correlation of rs-fMRI time series. Finally, derivatives are normalized and smoothed.

### Network-based analysis

The network-based analysis was performed using the toolbox for Data Processing & Analysis for Brain Imaging on Network (DPABINet) (39) (DPABINet: A toolbox for brain network and graph theoretical analyses). The function and structure networks are constructed by function connection matrix and structure connection matrix.

### Statistical analysis

Statistical analyses of demographic and clinical data were analyzed with SPSS for windows (SPSS, Version 23, IBM Corp.). *A priori* power analysis indicated that with *n* = 44 per group, *α* = 0.05 (two-tailed), and effect size *d* = 0.80, statistical power exceeded 92% for detecting group differences in DTI/fMRI metrics. Independent two-sample *t*-test and the Mann– Whitney *U* test were used to analyze the continuous variables. Chi-squared test was used to test the categorical variables. For ALFF analysis, two-sample *t*-test was operated to detect the differences between the two groups. For both ALFF and FC analysis, *p* < 0.05 was set as the statistical threshold at the voxel level, and we used permutation test with family-wise error (FWE) correction while setting the statistical threshold of *p* < 0.05 at the cluster level. Subgroup analyses were performed separately with different sides (left or right), preoperative dysfunction (with or without), and preoperative seizure (with or without) as criteria. Two-tailed statistical significance was set at *p* < 0.05.

## Results

### Baseline characteristics

Our study cohort comprised 44 individuals with AVMs, with a mean age of 33.00 ± 12.40 years, and 54.55% of them were male. There were 14 eloquent AVMs (32%). The mean volume of AVMs is 126.6 (95% CI: 70.1–257.5) mm^3^. All participants in this study were right-handed. There are 45% of AVMs had neurological dysfunction and 40.91% of patients had epilepsy, which was significant difference from NC (*p* = 0.002, *p* < 0.001, respectively).

### Brain functional alterations

**Figure 1:**
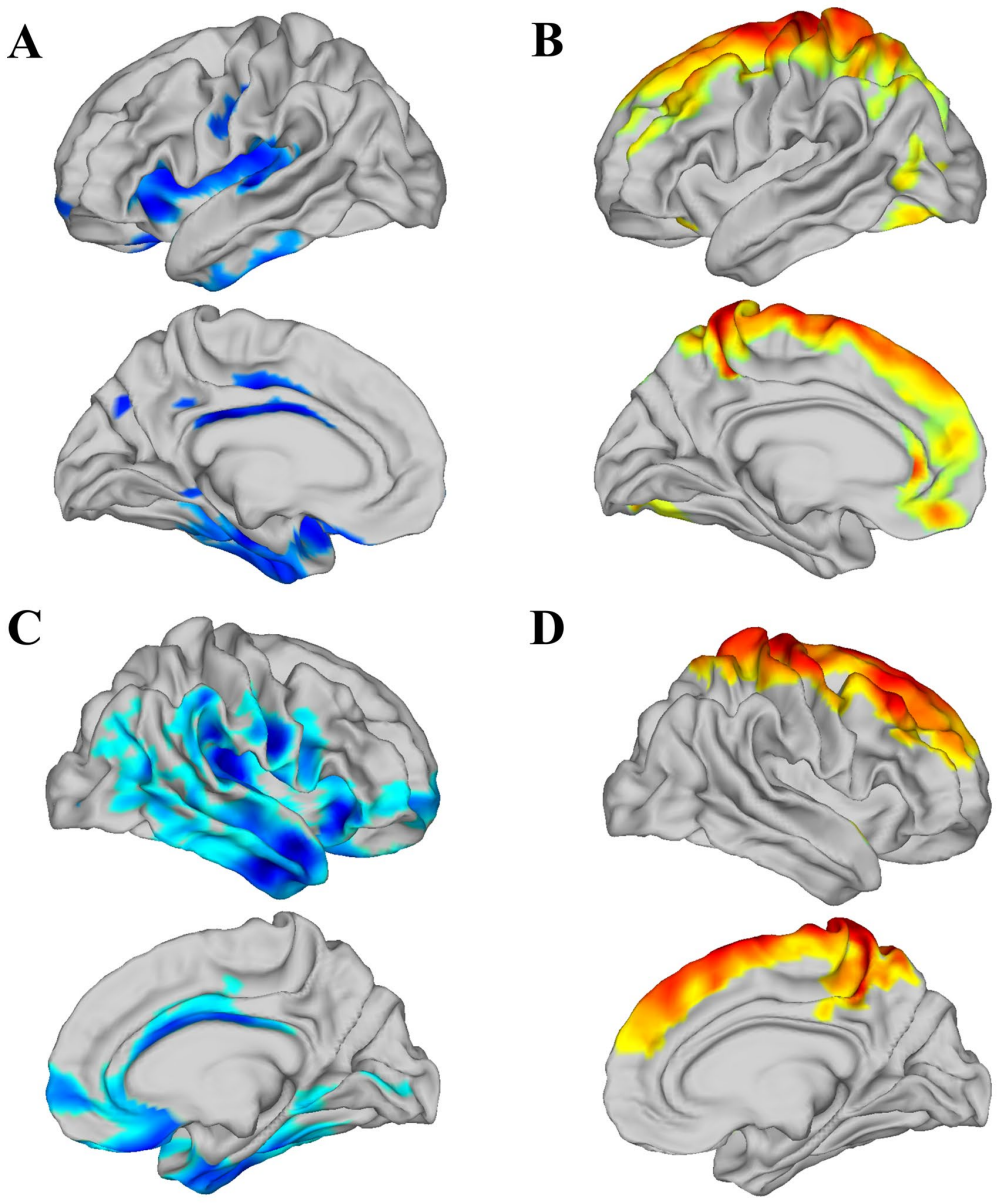
There are significant differences in the ALFF values of the cortical hemispheres between PT and HC (*p* < 0.05). **A** represents the region of decreased ALFF in the left hemisphere, while **B** represents the region of increased ALFF in the left hemisphere. **C** denotes the region of decreased ALFF in the right hemisphere, and **D** denotes the region of increased ALFF in the right hemisphere.

**Figure 2:**
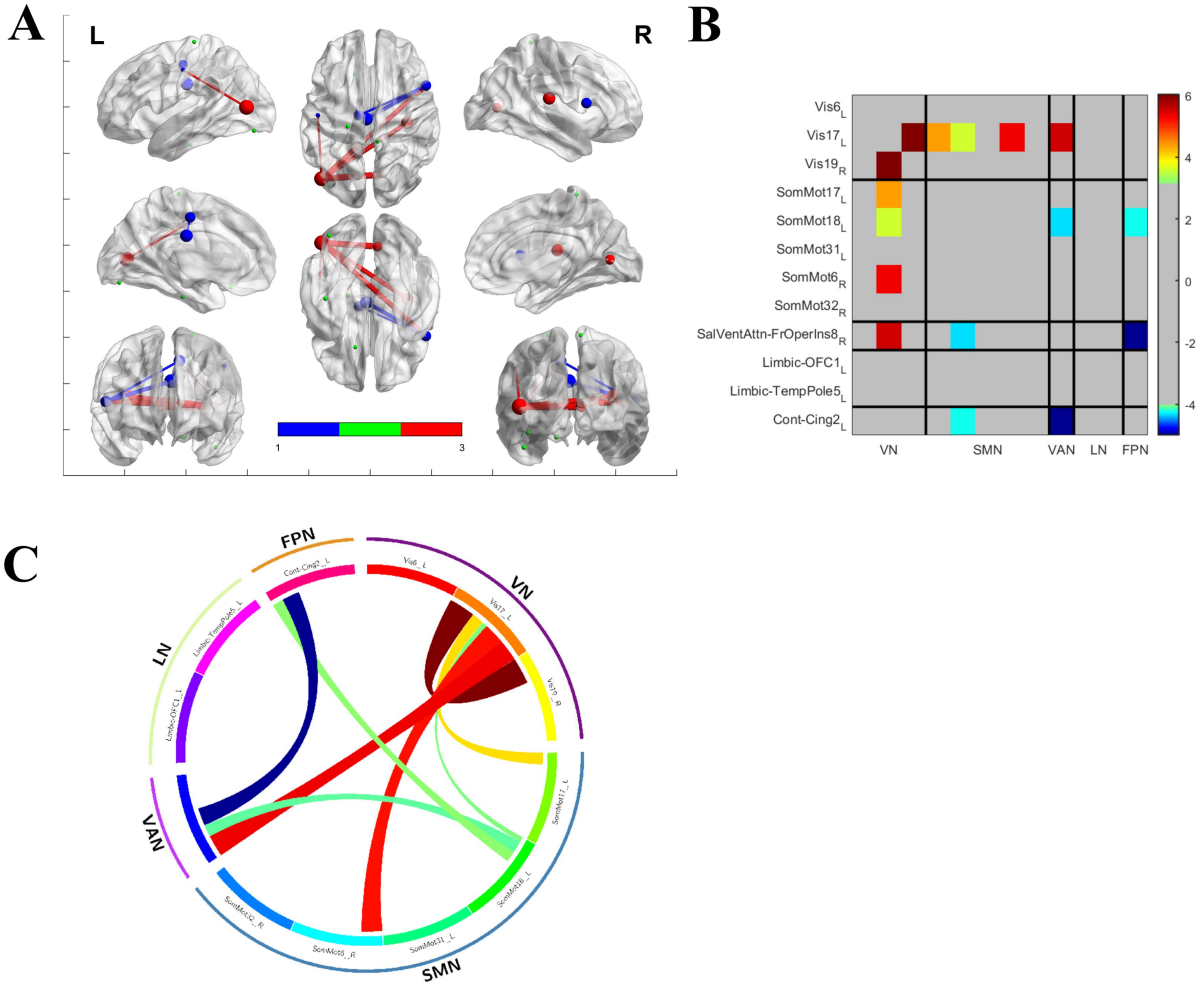
There are changes in brain functional connectivity strength between PT and HC **(A)**, with the alterations mainly occurring in the Visual Network (VN) and Somatomotor Network (SMN) **(B)**. In addition to the increased functional connectivity between VN and SMN, decreased functional connectivity of the Ventral Attention Network (VAN) with other networks can also be observed **(C)**.

Compared to HC, AVMs exhibited significant alterations in ALFF across 12 of the 400 brain regions defined by the Scheafer 400 atlas (40) (Table 1; Figure 1). The functional connectivity network, mapped with these regions as regions of interest, unveiled significant differences in connectivity between AVMs and HC (*p* < 0.05) (Figures 2A,B). Compared to HC, AVMs showed enhanced connectivity between the VN and other networks, and reduced connectivity of the VAN network with all networks (Figure 2C).

**Table 1:** Differences in ALFF between the PT and HC groups.

Brain region	Location	Network	Peak *T* value	Result
LH_SomMot_31	Left dorsal postcentral gyrus	SMN	11.47	PT > HC
LH_Vis_6	Left posterior lingual gyrus	VN	5.31	PT > HC
LH_Vis_17	Left posterior fusiform gyrus	VN	4.60	PT > HC
RH_SomMot_32	Right medial dorsal postcentral gyrus	SMN	10.53	PT > HC
LH_Limbic_TemPole_5	Left anteromedial temporal pole	LN	−7.10	PT < HC
LH_SomMot_17	Left ventral precentral gyrus	SMN	−8.04	PT < HC
LH_Cont_Cing_2	Left dorsal anterior cingulate cortex	FPN	−7.52	PT < HC
LH_SomMot_18	Left mid postcentral gyrus	SMN	−5.91	PT < HC
LH_Limbic_OFC_1	Left ventromedial orbitofrontal cortex	LN	−5.35	PT < HC
RH_SomMot_6	Right mid precentral gyrus	SMN	−10.33	PT < HC
RH_SalVentAttn_FrOper_8	Right frontal operculum	VAN	−5.63	PT < HC
RH_Vis_19	Right mid fusiform gyrus	VN	−3.60	PT < HC

FWE *p* < 0.05, cluster size ≥100 mm^2^.

FWE, Family-Wise Error correction; MNI, Montreal Neurological Institute template; PT, Patients; HC, Healthy Controls.

### Brain fiber alterations

**Figure 3:**
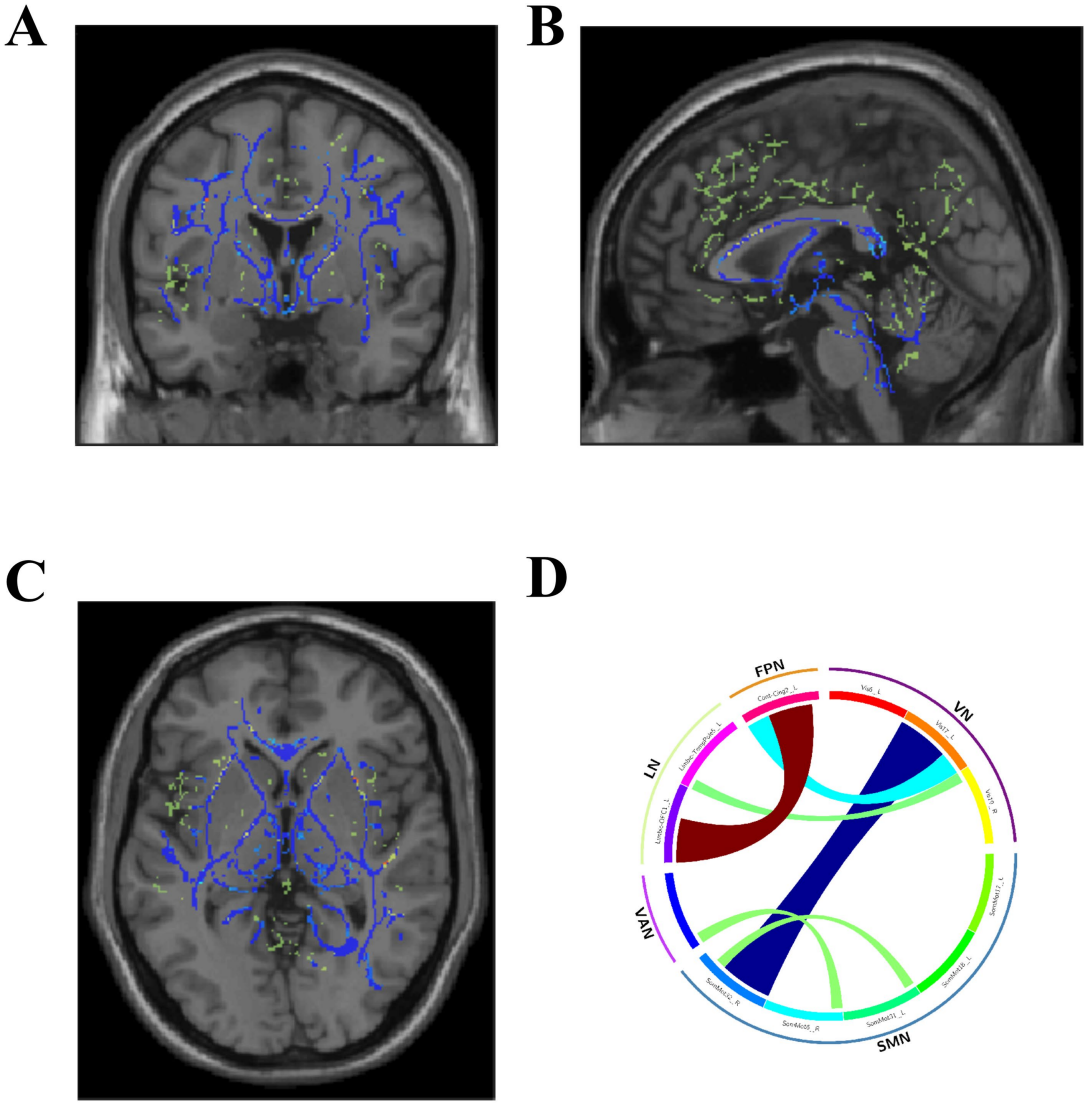
FA differences between PT and HC can be observed in the coronal **(A)**, sagittal **(B)**, and axial **(C)** views, primarily showing a decrease. The fiber connectivity matrix shows weakened connections between the Visual Network (VN) and the Somatomotor Network (SMN) **(D)**.

**Figure 4:**
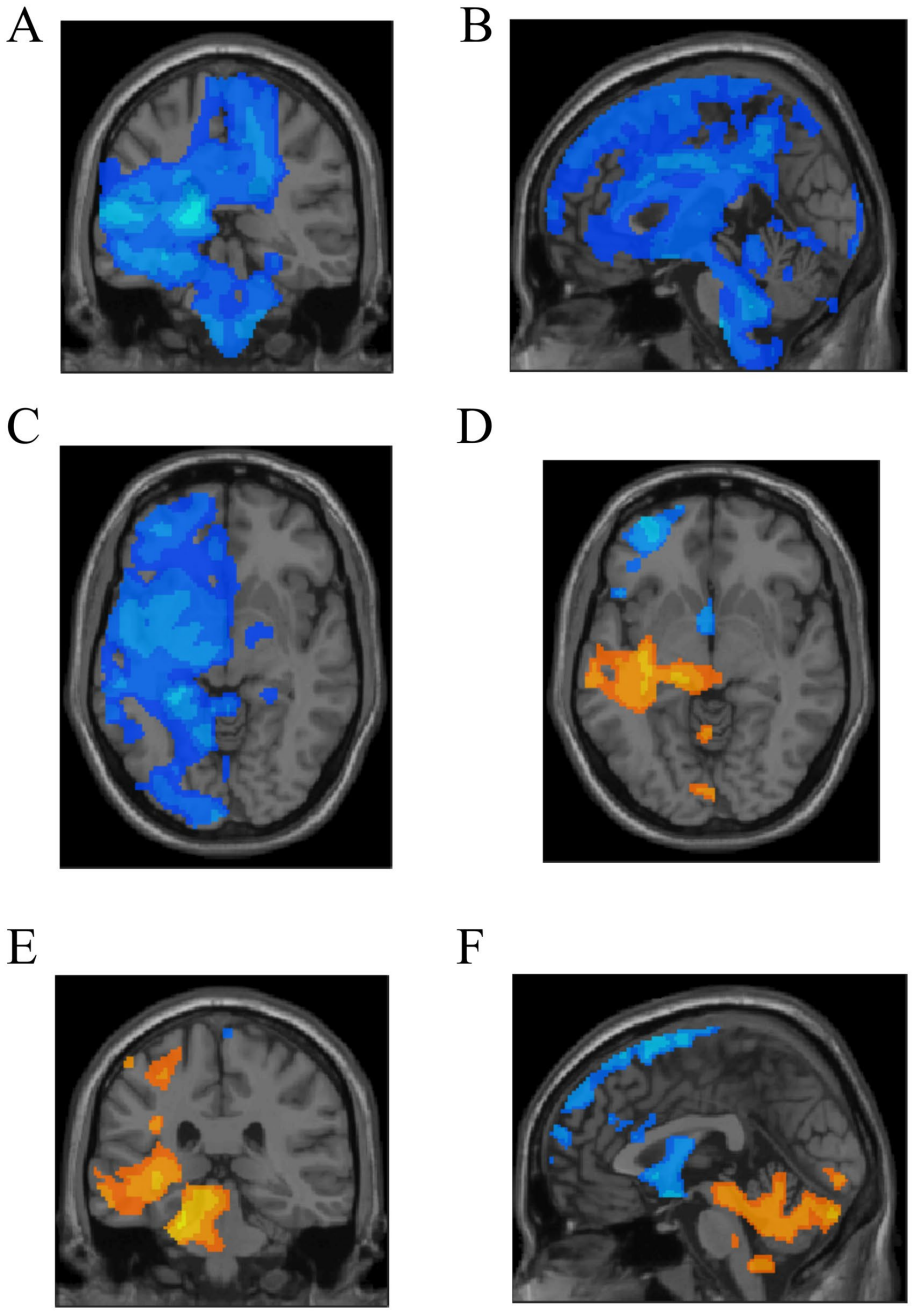
In the PT group, only fiber density decreases were observed in the fiber tracts passing through four ROIs, as shown in **A–C**. For the remaining ROIs, the fibers exhibited both increases and decreases in TDI **(D–F)**.

In the AVMs, FA was significantly reduced compared to NCs (*p* < 0.05) (Figures 3A–C) indicating a compromised structural alternation of white matter tracts. The fiber connectivity matrix indicated reduced fiber connections between the VN and the SMN (Figure 3D). An analysis of fiber connection density across 12 ROIs revealed significant differences between the two groups. Among these, 4 ROIs showed a significant reduction in track density imaging (TDI) between the two groups (Table 2; Figures 4A–C), whereas other regions depicted a mixed pattern of increased and decreased density (Figures 4D–F) (*p* < 0.05).

**Table 2:** ROIs with only decreased TDI.

ROI	Results
LH_SomMot_17	PT < HC
LH_Cont_Cing_2	PT < HC
RH_SomMot_32	PT < HC
RH_SalVentAttn_FrOper_8	PT < HC

### TW-FC changes

**Figure 5:**
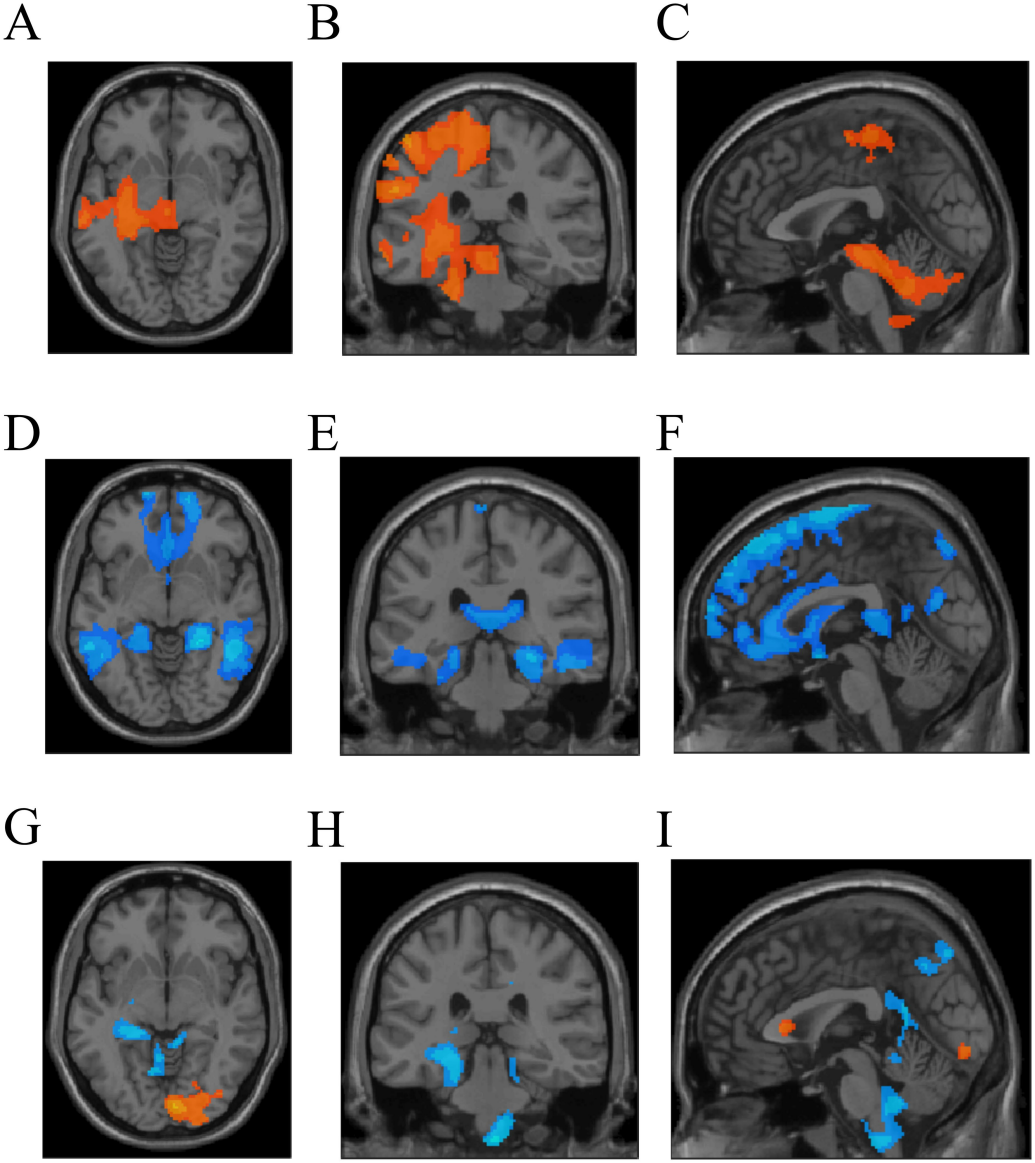
In 4 ROIs, TW-FC showed either solely increases **(A–C)** or decreases **(D–F)**. The remaining ROIs exhibited both increased and decreased connectivity nodes **(G–I)**.

The TW-FC analysis unveiled significant variations across the 12 ROIs when comparing AVM patients to HCs. In AVMs, 4 ROIs exhibited either only increased or only decreased TW-FC (Figures 5A–F; Table 3), while the remaining ROIs showed a bidirectional shift, with both enhancements and reductions noted (Figures 5G–I) (*p* < 0.05). In all ROIs, the functional connectivity strength of the traversing fiber tracts was altered.

**Table 3:** ROIs with only increased or decreased TW-FC.

ROI	Results
LH_SomMot_17	PT > HC
LH_Limbic_OFC_1	PT < HC
LH_SomMot_31	PT < HC
RH_SomMot_6	PT < HC

### Subgroup analysis

Subgroup analysis showed that these 4 ROIs exhibited TW-FC level changes in AVMs of different subgroups, and most of the ROI changes were consistent with our findings in the overall cohort (Supplementary Table S1; *p* < 0.05). A few ROIs showed different trends among groups, which may be related to the small sample size of each subgroup.

## Discussion

Our findings reveal concurrent alterations in white matter integrity, characterized by functional network reorganization. Previous research has established that arteriovenous malformations (AVMs) can affect deep white matter fiber tracts within eloquent areas (9, 10), even if the lesions are not located in these areas (41). Our study extends this understanding by demonstrating concurrent functional and structural alterations associated with AVMs—a dimension often overlooked in prior investigations. The simultaneous changes we observed in cortical function and fibers, along with alterations in TW-FC (time-varying functional connectivity), suggest that AVMs modify key structural nodes within functional connectivity pathways. These findings align with reports of functional reorganization following AVM resection, where the recruitment of homologous regions and changes in white matter tracts were pivotal for post-surgical language recovery (42). Several studies have reported altered functional and structural connectivity in AVMs. Using fMRI and DTI, Liu et al. observed significant functional reorganization following AVM resection, with recruitment of the right hemispheric homologous regions of Broca’s and Wernicke’s areas and changes in related white matter tracts. This reorganization was crucial for language recovery post-surgery (42). A study on AVMs located in Broca’s area utilized diffusion imaging and graph theory to analyze structural brain networks (43). The findings indicated altered topological properties, such as connectivity strength and efficiency, in patients compared to controls. These changes highlight how AVMs can disrupt the structural connectivity of critical language regions, leading to potential functional impairments. But above studies have not integrated these modalities to examine how structural changes contribute to functional reshaping. Our work represents one of the first comprehensive analyses of both functional and fiber reconstruction in AVMs. Using TW-FC analysis, we provided novel evidence that AVM-related changes in the visual network (VN) and sensorimotor network (SMN) may reflect the brain’s attempt to reorganize function through fiber tract modification. Preoperative TW-FC mapping revealing networks exhibiting significant hyperconnectivity or altered TW-FC may assist clinically identifying “at-risk” regions potentially heavily reliant on compensatory mechanisms. Surgical or radiosurgical interventions targeting AVMs embedded within or adjacent to these networks may necessitate more aggressive functional mapping and preservation procedures (e.g., intraoperative electrophysiological monitoring) to mitigate the risk of postoperative complications. Future studies must correlate TW-FC metrics with standardized neuropsychological testing and long-term clinical outcomes to establish their predictive value and specificity. Therefore, TW-FC holds potential as a more sensitive biomarker for identifying AVM-induced functional or structural alterations; however, robust validation in larger, prospectively designed cohorts remains essential prior to clinical implementation.

We found simultaneous alterations in both function and fibers within the VN and SMN in AVMs. However, only a minority of patients exhibited clinical presentations of neurological functional deficit. Reports indicate that less than a quarter of AVMs experience progressive neurological deficits (44, 45), which aligns with our findings. This also aligns with animal models showing lesion-induced myelin remodeling facilitates functional rewiring (46), and in humans, such gradual plasticity may enable functional preservation during slowly evolving lesions. When comparing patients with functional impairments to other patients, we found only one additional brain region with decreased function that was not among the previously identified 12 ROIs (Supplementary Table S2; Supplementary Figure S1). This suggests that changes in these two brain networks are not directly responsible for functional impairments. One explanation is that damage to connected cortical areas and along fiber tracts leads to hyperconnectivity and reorganization (47). Further, enhanced functional connectivity was also observed in the SMN in our study. Considering the relatively few local neurological symptoms in AVMs, this functional hyperconnectivity may be a compensatory brain strategy to offset functional deficits (48, 49). During recovery, disinhibition phenomena may occur (50), and the enhanced cortical signals could reflect cortical disinhibition and neuroplasticity. Finally, the bidirectional TW-FC shifts may indicate pathological network destabilization rather than beneficial reorganization, as seen in other neurological disorders (51). These alternative interpretations highlight the multifactorial complexity of AVM-related brain changes and emphasize the need for longitudinal multimodal studies to disentangle these possibilities. Without neuropsychological testing, we cannot confirm whether these connectivity changes genuinely improve functional output. Importantly, increased synchrony may represent maladaptive reorganization rather than beneficial compensation, as similar hyperconnectivity patterns precede pathological states like epilepsy (52). Therefore, we propose these alterations represent potential adaptive responses, acknowledging that the clinical significance requires validation through behavioral correlation. Additionally, the dominant hemisphere was previously found receives relatively less interhemispheric inhibition from the non-dominant hemisphere (53). Our study also found that cortical ALFF changes were greater in the dominant hemisphere.

Cortical functional changes may play a crucial role in AVM-related epilepsy (54–56). Epilepsy is a common symptom among AVMs (22.7–60.9%) (57–59). In this study, we observed a significant decrease in cortical ALFF values in AVMs presented with seizures (*p* < 0.05) (Supplementary Figure S2). Currently, there are conflicting findings regarding whether ALFF value changes in epileptic brains are increased or decreased (54, 56, 60). Some studies posit that hyperactivity could be a compensatory mechanism or a result of abnormal synchronization within the brain networks. Conversely, other research indicates decreased ALFF values, suggesting a possible reduction in neural activity due to impaired functional connectivity or neuronal loss in regions affected by seizures. Based on the results of this study, we hypothesize that epilepsy in AVM patients may be caused by local functional connectivity disruptions that arise during the process of functional reorganization. Further research is essential to understand how these networks’ functional and structural integrity is compromised in AVM patients with epilepsy, which may reveal new insights into the pathophysiology of seizures in AVM patients and help identify potential therapeutic targets.

In this study, we visualized the modifications of fiber tracts using DTI while previous studies have discussed the relationship between fiber tracts affected by AVMs and clinical symptoms (13, 14), our study is the first to combine Resting-state fMRI and DTI to investigate AVM-induced changes. AVMs can alter surrounding fiber tracts, which is related to their pathophysiological behavior, including bleeding and ongoing dynamic evolution (61). Not only functional reshaping but also reconstruction of fiber tracts near AVMs has been observed (22), consistent with the results of our study. Additionally, as congenital lesions, AVMs may alter neurodevelopmental trajectories, making it difficult to distinguish between true reorganization and innate neuroanatomical differences (58). However, due to the limited number of AVMs, we were unable to further discuss the relationship between AVMs and nearby fiber reconstruction and functional changes based on the location of the AVMs. Larger-scale, multicenter studies may be needed in the future to discuss the impact of AVMs on fiber tracts more comprehensively.

However, our study also has limitations. Firstly, the healthy controls in our study were obtained from a public database, which may introduce biases due to factors such as equipment, center, ethnicity, etc., reducing the robustness of our conclusions. Different scanning devices in different centers may lead to the bias of ALFF and FA. Educational attainment (unavailable in public datasets) correlates with functional connectivity strength, potentially confounding network-level comparisons. These limitations need cautious interpretation of absolute effect sizes. Future multicenter studies with standardized protocols are essential to confirm these results. Secondly, the relatively small patient cohort hindered classification based on clinical data such as lesion location, lesion size and patient symptoms, impeding further exploration of AVM-related changes in function and structure. Future studies are essential to discuss these characters in a larger cohort. Lastly, we did not use scales to assess the cognitive abilities of patients, which may lead to an underestimation of clinical symptoms related to brain function in patients.

## Conclusion

In summary, our study revealed a profound association between AVMs and a spectrum of alterations in both brain function and fiber reorganization. These changes extend beyond focal regions, reverberating across neural networks to significantly reshape functional connectivity in key hubs. We observed concurrent functional and fiber connectivity changes between the VN and SMN in AVMs, suggesting the brain’s innate capacity to marshal compensatory mechanisms. Furthermore, concurrent functional and structural connectivity changes between the visual network VN and SMN suggest the innate capacity to deploy compensatory mechanisms of brain. Our works paved the way for future research dissecting the interplay between AVMs and brain plasticity and emphasize the need for nuanced approaches. Future studies should correlate AVM neurofunctional and structural features with clinical symptomatology to broaden the utility of neurofunctional and structural biomarkers in AVM management.

## Data Availability

The raw data supporting the conclusions of this article will be made available by the authors, without undue reservation.
