# Supplementary material for: Concurrent functional-structural reorganization in brain networks of AVM patients: a functional and structural study
Source: Front Neurol. 2025 Oct 28;16:1619226. doi: 10.3389/fneur.2025.1619226 (PMC12604527; doi:10.3389/fneur.2025.1619226)
Supplement: Supplementary file 1 [file Table_1.docx]

**Table S1** Subgroup analysis of ROIs exhibiting TW-FC unidirectional variation

| ROI | Results |
| --- | --- |
| 1. AVMs located in left cerebral hemisphere | |
| LH_SomMot_17 | PT>HC |
| LH_Limbic_OFC_1 | PT<HC |
| LH_SomMot_31 | PT<HC |
| RH_SomMot_6 | PT<HC |
| 2. AVMs located in right cerebral hemisphere | |
| LH_SomMot_17 | PT>HC |
| LH_Limbic_OFC_1 | PT<HC |
| LH_SomMot_31 | PT<HC |
| RH_SomMot_6 | PT<HC |
| 3. AVMs with epilepsy |  |
| LH_SomMot_17 | PT>HC |
| LH_Limbic_OFC_1 | PT<HC |
| LH_SomMot_31 | PT<HC |
| RH_SomMot_6 | PT<HC |
| 4. AVMs without epilepsy |  |
| LH_SomMot_17 | PT>HC |
| LH_Limbic_OFC_1 | PT<HC |
| LH_SomMot_31 | PT<HC |
| RH_SomMot_6 | PT<HC |
| 5. AVMs with neurological dysfunction |  |
| LH_SomMot_17 | - |
| LH_Limbic_OFC_1 | - |
| LH_SomMot_31 | PT<HC |
| RH_SomMot_6 | - |
| 6. AVMs without neurological dysfunction |  |
| LH_SomMot_17 | PT>HC |
| LH_Limbic_OFC_1 | PT<HC |
| LH_SomMot_31 | PT<HC |
| RH_SomMot_6 | PT<HC |
