# Supplementary material for: Concurrent functional-structural reorganization in brain networks of AVM patients: a functional and structural study
Source: Front Neurol. 2025 Oct 28;16:1619226. doi: 10.3389/fneur.2025.1619226 (PMC12604527; doi:10.3389/fneur.2025.1619226)
Supplement: Supplementary file 2 [file Table_2.docx]

Table S2 Differences in ALFF between AVM patients with functional impairments and those without functional impairments.

| Brain region | MNI peak coordinates | | | Cluster Size (Vertices) | *T* value | Result |
| --- | --- | --- | --- | --- | --- | --- |
|  | X | Y | Z |  |  |  |
| LH_Vis_26 | -20.9589 | -68.1492 | 16.7083 | 1229 | -4.43233 | With functional impairments <Without |
