# Supplementary figures and images for: Concurrent functional-structural reorganization in brain networks of AVM patients: a functional and structural study
Source: Front Neurol. 2025 Oct 28;16:1619226. doi: 10.3389/fneur.2025.1619226 (PMC12604527; doi:10.3389/fneur.2025.1619226)

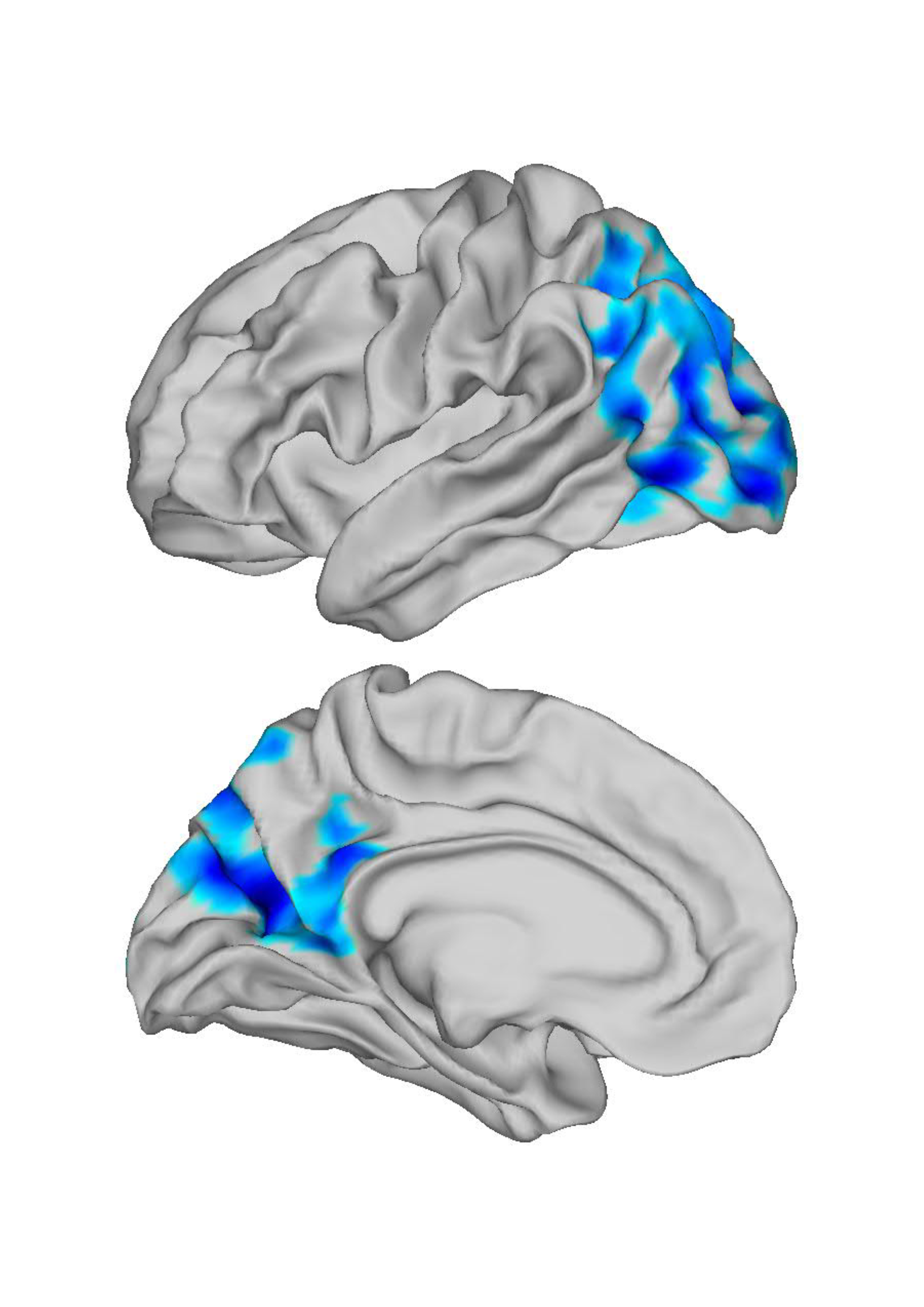

Supplement: Supplementary file 3 [file Image_1.tif]

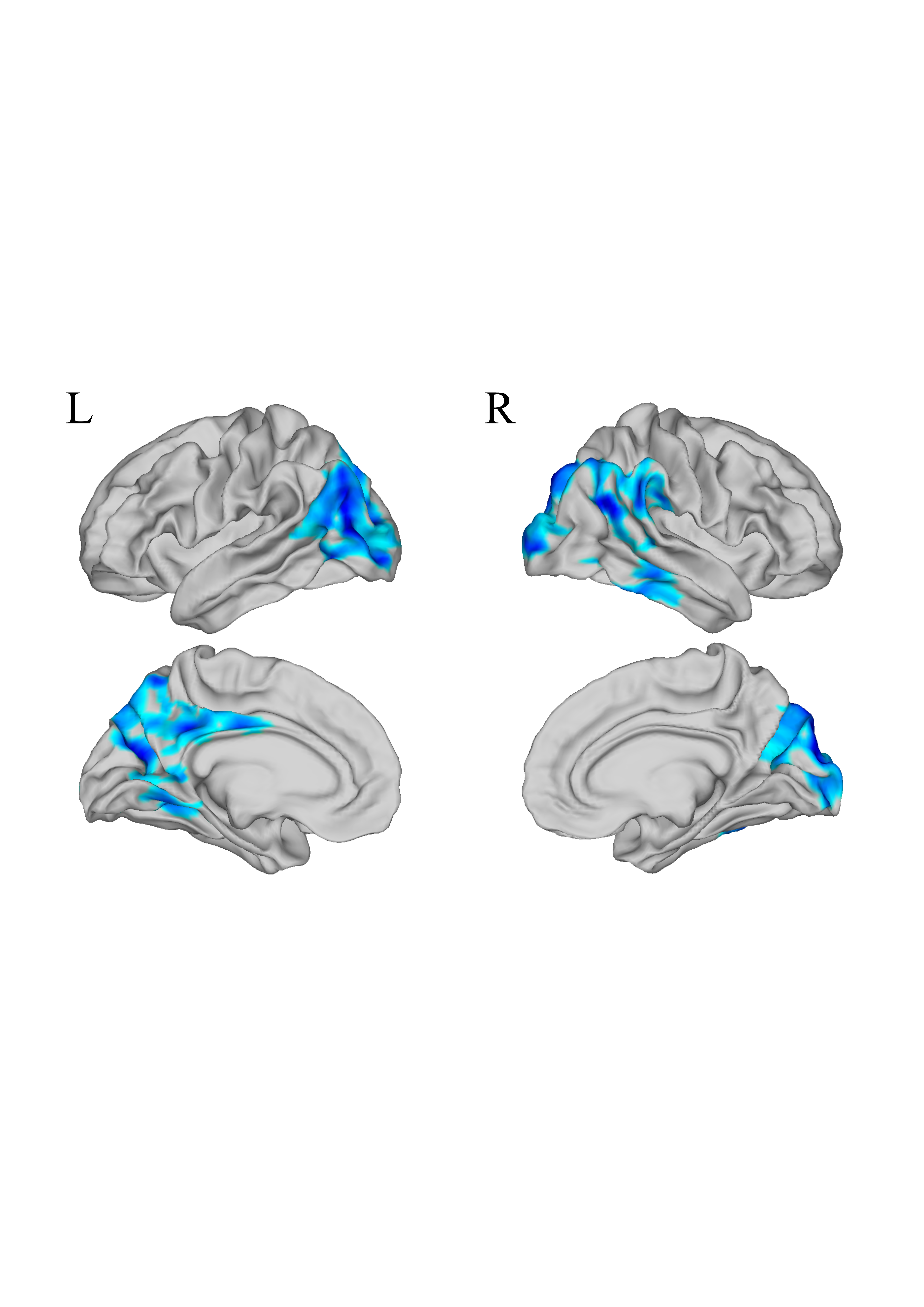

Supplement: Supplementary file 4 [file Image_2.tif]
